# The causal effect of smoking on psychiatric disorders: an examination of brain volume as a potential pathway

**DOI:** 10.1017/S0033291725100561

**Published:** 2025-06-24

**Authors:** Margot P. van de Weijer, Shu Liu, Anaïs B. Thijssen, Robyn E. Wootton, Adrià Túnez, Jentien M. Vermeulen, Guido van Wingen, Dirk J. A. Smit, Marcus Munafò, Karin J. H. Verweij, Jorien L. Treur

**Affiliations:** 1 Department of Psychiatry, Amsterdam UMC, University of Amsterdam, Amsterdam, the Netherlands; 2 Amsterdam Public Health Research Institute, Amsterdam University Medical Centre, Amsterdam, the Netherlands; 3 School of Psychological Science, University of Bristol, Bristol, UK; 4 Nic Waals Institute, Lovisenberg Diaconal Hospital, Oslo, Norway; 5 Amsterdam Brain and Cognition, University of Amsterdam, Amsterdam, the Netherlands

**Keywords:** brain volume, genetics, genomic SEM, Mendelian randomization, psychiatry, smoking

## Abstract

**Background:**

There is growing evidence that smoking increases the risk of developing psychiatric disorders, but the underlying mechanisms are largely unknown. We examine brain structure as a potential pathway between smoking and psychiatric disease liability.

**Methods:**

We test associations between smoking (initiation, cigarettes per day, cessation, lifetime use) and depression, bipolar disorder, and schizophrenia, with and without correcting for volume of the amygdala, hippocampus, lateral and medial orbitofrontal cortex, superior frontal context, and cortical thickness and surface area. We use three methods that use summary statistics of genome-wide association studies to investigate genome-wide and local genetic overlap (genomic structural equation modeling, local analysis of (co)variant association), as well as causal associations (Mendelian randomization).

**Results:**

While we find causal effects of smoking on brain volume in different brain areas, and with psychiatric disorders, brain volume did not seem to mediate the effect of smoking on psychiatric disorders.

**Conclusions:**

While these findings are limited by characteristics of the included summary statistics (e.g. sample size), we conclude that brain volume of these areas is unlikely to explain a substantial part of any effect of smoking on psychiatric disorders. Nevertheless, genetic methods are valuable tools for exploring other potential mechanisms, such as brain functional connectivity, foregoing the need to collect all phenotypes in one dataset.

## Introduction

Smoking is one of the leading preventable risk factors for morbidity and mortality (Effertz & Karl, 2013). It is much more common among individuals with psychiatric disorders, such as major depressive disorder and schizophrenia (Talati, Keyes, & Hasin, 2016), who – likely because of this – have a lower life expectancy (Hjorthøj, Stürup, McGrath, & Nordentoft, 2017; Rosoff et al., 2024). Previous research suggests bidirectional causal effects between smoking and psychiatric disorders (Taylor & Treur, 2023). The causal effect of smoking on psychiatric outcomes is especially clinically relevant, as this suggests that smoking cessation may have mental health benefits in patient populations. While this is of major public health relevance, there is a surprising lack of clarity regarding the mechanisms that may underlie this pathway.

There are different lines of evidence revealing a causal effect of smoking on psychiatric outcomes. A large systematic review by Taylor and colleagues that included longitudinal studies of any clinical population that report change in depression, anxiety, positive affect, psychological quality of life or stress from a pre-quit attempt to follow-up (6 weeks later) indicated that smoking cessation is associated with later improvements in mental health (Taylor et al., 2021). When performing adjusted analyses where common confounders such as age, sex, education, and income were included, no meaningful change in results was observed, showing that the effect is unlikely to be confounded by these factors. In addition, 56 of the 102 studies performed secondary analyses of randomized controlled trials, indicating that reverse causation is unlikely to bias these results. In addition, a systematic review of Mendelian Randomization studies (MR, a form of instrumental variable analysis where genetic variants are included as instruments, explained in more detail in the methods section) found evidence for bidirectional causal effects of smoking on depression, bipolar disorder and schizophrenia (Treur, Munafò, Logtenberg, Wiers, & Verweij, 2021).

There are likely different environmental and (neuro)biological pathways that underlie the smoking-psychiatric disease associations. On the environmental side, it may be the case that factors such as socioeconomic status (SES) mediate or confound these associations. For example, it is possible that individuals with low SES are both more likely to smoke and more likely to develop a psychiatric disorder (with current literature providing mixed results for this). Similarly, there are different neurobiological mechanisms that (together) may explain effects of smoking on risk of psychiatric disorders, such as inflammation, alterations in neural functional connectivity, or reduced neurogenesis (van de Weijer et al., 2024). Nicotine can modulate neurotransmitter activity of many different neurotransmitters (e.g. dopamine, serotonin, acetylcholine, glutamate) by binding to nicotinic acetylcholine receptors distributed widely across the central nervous system. Through this neurotransmitter activity, nicotine has been found to affect different cognitive processes, such as attention, reward, and motivation – processes that are also affected in different psychiatric disorders (Chau, Roth, & Green, 2004; Quattrocki, Baird, & Yurgelun-Todd, 2000).

A potential long-term mechanism that has received little attention so far is changes in the structure or volume of certain brain areas. Many imaging studies have linked smoking behaviors to differences in brain volumes (Gray et al., 2020; Zhong et al., 2016). For example, imaging meta-analyses have found volume differences between smokers and non-smokers in areas of the (pre)frontal cortex, insula, and cingulate cortex (van de Weijer et al., 2024), and studies in rats have shown that nicotine damages developing brain cells in a way that interferes with cell replication and differentiation and can evoke apoptosis (Slotkin, 2002). A recent study provided compelling evidence that smoking can causally decrease subcortical volume, specifically in the amygdala and hippocampus, by using genetic variants associated with smoking as genetic instruments through MR, outlined below in more detail (Logtenberg et al., 2022). Similarly, there is considerable evidence for altered brain volumes in patients with bipolar disorder (Bora, Fornito, Yücel, & Pantelis, 2010; Wang et al., 2019), major depression (Brandl et al., 2022; Schmaal et al., 2016; Sexton, Clare, & Ebmeier, 2013), and schizophrenia (Shah et al., 2017; Vita, De Peri, Deste, & Sacchetti, 2012). Examples are frontal areas such as the medial and lateral orbitofrontal and superior frontal cortex (Bora et al., 2011; Lu et al., 2019; Wise et al., 2017), areas that have also been implicated in smoking (Sutherland et al., 2016; Yang, Zhang, Cheng, & Zheng, 2020; Zhong et al., 2016). Combining these insights leads to the hypothesis that causal effects of smoking on the liability for developing psychiatric disorders may be mediated by structural changes in the brain. Although this causal chain seems plausible, it has not yet been formally tested.

Studying smoking to brain volume to psychiatric disorder pathways is difficult because it requires large imaging datasets in individuals with (and without) these disorders, including information on their smoking status. In addition, obtaining causal evidence is difficult since we cannot ethically perform a randomized controlled trial where we allocate individuals to a smoking ‘treatment’. However, an alternative way in which we can study these pathways is by using genetic data. There are different methods available that employ genetic information to get better insight into causal pathways between two or more variables of interest. The advantage of these methods is that the variables in question do not necessarily need to be available in the same dataset, as long as the samples are drawn from similar populations. For example, it is possible to calculate a genetic correlation (quantification of genetic overlap) between two sets of summary statistics from separate genome-wide association studies (GWASs). Using these genetic methods, we aim to examine and distinguish so-called horizontal pleiotropy from vertical pleiotropy. With horizontal pleiotropy, the same genes influence multiple traits, thus inducing an association between the two even in the absence of a causal association (‘genetic confounding’). With vertical pleiotropy, genes directly associated with an exposure become indirectly associated with an outcome (and potential mediators of the association) through the causal chain from the exposure to the outcome. In this way, these genetic methods can be used to study the presence of potential mechanisms without these necessarily reflecting a genetic mechanism. For this purpose, we use three methods.

First, genomic structural equation modeling (GSEM) (Grotzinger et al., 2019) uses effect size estimates from genetic variants (from existing GWASs) across the whole genome to model complex multivariable relationships. We use this method to establish if there is genetic pleiotropy between the traits, which may be due to both horizontal and vertical pleiotropy. Second, a method for examining genetic associations on a more detailed genomic level is local analysis of (co)variant association (LAVA) (Werme, Van Der Sluis, Posthuma, & De Leeuw, 2022). LAVA can be used to find out if *local* (i.e. specific regions of the genome) genetic correlations between phenotypes (e.g. smoking and major depression) are mediated by other phenotypes (e.g. cortical surface area). We include this method to examine the presence of local genetic correlations, which may exist even with a low or absent global genetic correlation (and could thus cancel each other out on a global level). Finally, MR can formally test *causal* effects by employing genetic variants predictive of an exposure as instrumental variables. The method can be extended to assess the independent direct effects of multiple exposures through multivariable MR (Burgess & Thompson, 2015). This analysis goes a step further than the genetic correlation analyses by (through sensitivity analyses) trying to distinguish horizontal (confounding) from vertical (causal) pleiotropic effects.

This is the first study testing if structural brain measures mediate the association between smoking phenotypes and psychiatric disease (bipolar disorder, major depression, schizophrenia). Based on the prior evidence, we focus on volume of the amygdala and hippocampus, the lateral and medial orbitofrontal cortex, and the superior frontal cortex. We acknowledge that there are other brain areas that have previously been implicated in substance use and/or psychiatric disease. However, we are restricted to GWAS that include UK Biobank (UKB) data, since this is the largest imaging dataset that also includes genetic data. In prior work, we found that other than the amygdala and hippocampus, none of the subcortical structures in UKB were significantly causally associated with psychiatric disease, and thus these were left out. We additionally include frontal areas since these have specifically been implicated in both substance use and psychiatric disease (Koster et al., 2025; van de Weijer et al., 2024). In the UKB, volume measures of the following brain areas are available: caudal middle frontal, frontal pole, lateral OFC, medial OFC, rostral middle frontal, and superior frontal. To limit the number of tests, we include only OFC volume and superior frontal volume. In addition, we refrain from relying solely on specific genomic regions based on prior knowledge and additionally include measures of cortical surface area and thickness. We seek to clarify the plausibility of these global and regional differences as an underlying mechanism in the association between smoking and the risk of psychiatric disorders by studying potential mediation across three methods (GSEM, LAVA, and MR).

## Methods

This study was pre-registered at the Open Science Framework: https://osf.io/3eksj. Deviations from the pre-registration are listed in the Supplementary Methods.

### Summary data

We use summary statistics from large GWASs and meta-analyses. In a GWAS (Abdellaoui, Yengo, Verweij, & Visscher, 2023), associations between millions of genetic variants and an outcome are estimated. The resulting summary statistics are a list of these associations and their *p*-values. Detailed information on the summary statistics are provided in Table 1.

**Table 1.** Details of included GWAS summary statistics

Phenotype	Source	Sample	Measure
Smoking initiation	Liu et al. (2019)	European ancestry meta-analysis, including 23andme and excluding UKB (*N* = 848,460)	Binary phenotype: any participant reporting ever being a regular smoker in their life (current or former) were coded ‘2’, while any participant who reported never being a regular smoker were coded ‘1’. Was measured in three ways: a. Have you smoked over 100 cigarettes over the course of your life? b. Have you ever smoked every day for at least a month? c. Have you ever smoked regularly?
Cigarettes per day	Liu et al. (2019)	European ancestry meta-analysis, including 23andme and excluding UKB (*N* = 216,590)	Defined as the average number of cigarettes smoked per day, either as a current smoker or former smoker. For studies that collected a quantitative measure of cigarettes per day, responses were binned as follows: 1 = 1–5, 2 = 6–15, 3 = 16–25, 4 = 26–35, 5 = 36+
Smoking cessation	Liu et al. (2019)	European ancestry meta-analysis, including 23andme and excluding UKB (*N* = 630,154)	Binary phenotype with current smokers coded as ‘2’ and former smokers coded as ‘1’, and never smokers are coded as missing
Lifetime smoking	Wootton et al. (2020)	European ancestry UKB sample (*N* = 462,690)	Combines smoking status (current, former, never), age at initiation in years, age at cessation in years, time since cessation and number of cigarettes smoked per day into a lifetime smoking index along with a simulated half-life (τ) constant. Half-life captures the exponentially decreasing effect of smoking at a given time on health outcomes.
Hippocampus volume	Logtenberg et al. (2022)	European ancestry meta-analysis of ENIGMA and UKB (*N* = 50,290)	Sum of right and left brain volume (in mm^3^) as defined by structural MRI and corrected for total intracranial volume
Amygdala volume	Logtenberg et al. (2022)	European ancestry meta-analysis of ENIGMA and UKB (*N* = 50,290)	Sum of right and left brain volume (in mm^3^) as defined by structural MRI and corrected for total intracranial volume
Cortical surface area	Grasby et al. (2020)	European ancestry meta-analysis of ENIGMA and UKB (*N* = 33,992)	Whole cortex surface area derived from in vivo whole-brain T1-weighted MRI scan
Cortical thickness	Grasby et al. (2020)	European ancestry meta-analysis of ENIGMA and UKB (*N* = 33,992)	Thickness whole cortex derived from in vivo whole-brain T1-weighted MRI scan
Volume of the lOFC, mOFC and superior frontal cortex	UK Biobank	UKB GWAS (*N* = 36,443)	Sum of right and left brain volume (in mm^3^) as defined by structural MRI and corrected for total intracranial volume.
Major depression	Wray et al. (2018)	European ancestry meta-analysis, excl. 23andme and UKB (N_cases_ = 42,465, N_controls_ = 81,600, N_effective_ = 48,660)	Cases were required to meet international consensus criteria (DSM-IV, ICD–9, or ICD–10) for a lifetime diagnosis of major depressive disorder established using structured diagnostic instruments from assessments by trained interviewers, clinician-administered checklists, or medical record review.
Bipolar disorder	Mullins et al. (2021)	European ancestry meta-analysis, excl. UKB (*N*_cases_ = 40,463, *N*_controls_ = 313,436, _*N*effective_ = 48,144.46)	Cases were required to meet international consensus criteria (DSM-IV, ICD–9 or ICD–10) for a lifetime diagnosis of bipolar disorder, established using structured diagnostic instruments from assessments by trained interviewers, clinician-administered checklists or medical record review.
Schizophrenia	Trubetskoy et al. (2022)	European ancestry meta-analyses (*N*_cases_ = 53,386, *N*_controls_ = 77,258, *N*_effective_ = 58,749.13)	Cases were defined as individuals with schizophrenia, schizophrenia or schizoaffective disorder (SCZ/Schizoaffective), or schizophrenia spectrum disorder. Diagnostic strategy varies across cohorts, including consensus diagnosis, research diagnostic interview, review of medical records, and mixed strategy (see original paper for details).

*Note:* UKB, UK Biobank; lOFC, lateral orbitofrontal cortex; mOFC, medial orbitofrontal cortex.

As smoking exposures, we include European ancestry summary statistics for smoking initiation, cigarettes per day, and smoking cessation from Liu and colleagues (Liu et al., 2019) (including 23andme, Inc. and excluding UK Biobank to prevent overlap with the brain volume GWASs) and lifetime smoking (composite of smoking initiation, heaviness, duration and cessation) by Wootton and colleagues (Wootton et al., 2020). The summary statistics for smoking cessation are oriented so that higher scores reflect current smoking.

As mediators, we include European ancestry summary statistics for subcortical volume of the amygdala and hippocampus from Logtenberg and colleagues (2022), and surface area and thickness of the whole cortex from Grasby et al. (2020). For the MR analyses, we also employ the summary statistics for amygdala and hippocampal volume stratified for smoking status. Unfortunately, the cortical surface area and thickness summary statistics were only available for the full sample (unstratified for smoking). We ran (full and smoking-stratified) GWASs for lateral and medial orbitofrontal cortex and the superior frontal cortex ourselves in a subset of European ancestry individuals in UK Biobank (Bycroft et al., 2018) (see Supplementary Methods and Figures S1–S8).

As outcomes, we include European ancestry summary statistics for bipolar disorder (Mullins et al., 2021), major depressive disorder excluding 23andme (Wray et al., 2018), and schizophrenia (Trubetskoy et al., 2022) from the Psychiatric Genomics Consortium, excluding the UK Biobank for the MR analyses. These summary statistics are not available stratified for smoking.

### Analyses

#### Genomic SEM

**Figure 1.**
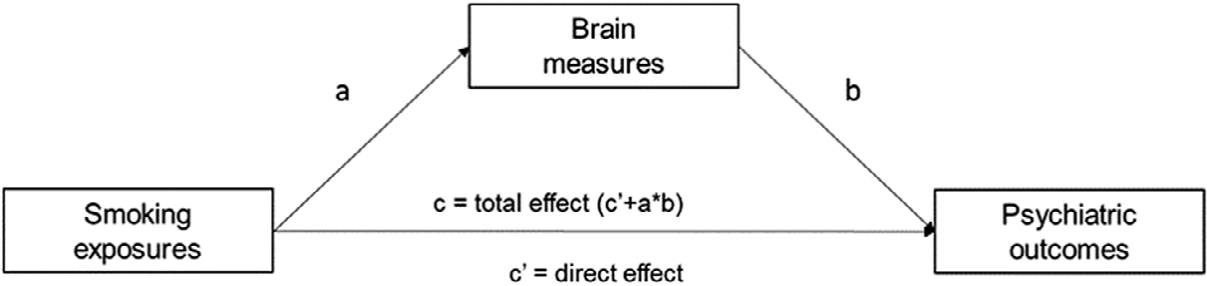
Path diagram assessing the brain measures as mediators of associations between different smoking exposures and psychiatric outcomes. The product of the mediation effect of the brain measures (a × b) and the direct effect of the smoking exposures (c’) is the total effect of smoking exposures on psychiatric outcomes (c’ + a × b).

We examine genome-wide overlap between the traits by calculating (partial) genetic correlations using the r-package GSEM. GSEM is an extension of LD score regression (Bulik-Sullivan et al., 2015) that can be used to model the multivariate genetic architecture of a set of traits (Grotzinger et al., 2019). Using GWAS summary data, GSEM allows for modeling user-specified structural models unbiased by sample overlap (Grotzinger et al., 2019). We use GSEM to calculate (partial) genetic correlations between smoking phenotypes and psychiatric outcomes while controlling for brain phenotypes (see Figure 1). The mediation/confounding (the method cannot distinguish between these) effect is calculated by multiplying the effect of association between the smoking exposures and the brain measures (a) with the effect of the association between the brain measures on the psychiatric outcomes (b). The product of the mediation effect and the direct effect of smoking (c) is the total effect and equals the bivariate genetic correlation.

#### LAVA

When there are local genetic correlations in opposite directions, they cancel each other out in global genetic correlations. We use LAVA (Werme et al., 2022) to calculate local genetic correlations between all smoking phenotypes and psychiatric outcomes. We first identify regions where both a predictor (smoking phenotype) and outcome (psychiatric disorder) exhibit significant SNP heritability for 2,495 regions based on region definitions by Werme and colleagues (Werme et al., 2022), using the 1000 Genomes European panel as a reference panel (MAF > .01) (Clarke et al., 2012). Significance of the SNP heritability is based on a significance threshold corrected for the number of studied regions (0.05/2,495 = .00002). For regions where both a predictor and outcome exhibit significant univariate signal, we run bivariate tests to identify local genetic correlations. Significance of the local genetic correlations is based on a significance threshold corrected for the number of regions examined in the bivariate analyses (0.05/758 = .000066). We repeat these analyses for associations between brain phenotypes and psychiatric outcomes. If a region shows significant correlations of a psychiatric outcome with a smoking phenotype and brain phenotype, we run multivariate regression models including both predictors in the model to assess potential mediation/confounding.

#### Mendelian randomization (MR)

We conduct MR to examine evidence for causal effects of liability to smoking on psychiatric disease risk and brain volume and of brain volume on psychiatric disease risk. MR can be seen as somewhat similar to a randomized controlled trial (RCT), but instead of randomization to a control and treatment group, individuals are randomized at conception with respect to which alleles they receive. Through this random allocation, we can distinguish groups of individuals with high lifetime genetic risk for an exposure from individuals with low lifetime genetic risk for an exposure, independent from potential confounders. Therefore, outcome differences between these groups must be due to differences in the exposure, dependent on some key assumptions. MR relies on the following assumptions: (1) the genetic variants (instruments) are robustly associated with the exposure, (2) the instruments are independent of population-level confounding, and (3) the instruments do not directly affect the outcome, only through the exposure. Inverse-variance weighted (IVW) regression implemented in the TwoSampleMR r-package (Hemani et al., 2018), is used as a main method of estimating causal effects. As instruments, we use harmonized independent genome-wide significant SNPs (*p* < 5×10^−8^) from the exposure GWASs. When this leads to the inclusion of five or fewer SNPs, we take a more liberal threshold of *p* < 1×10^−5^ (applied to amygdala volume, cortical thickness, and medial OFC volume). In IVW, a ratio estimate is obtained by dividing SNP-outcome effects by SNP-exposure effects. The individual SNPs are weighted by the inverse of their variance and combined to a single effect estimate. To assess instrument strength, we obtain an F-estimate and assess if *F* > 10 as a rule of thumb. Heterogeneity across the causal estimates of the SNPs in each instrument is assessed with Cochran’s Q-statistic. In addition, we perform leave-one-out (LOO) analyses, where we repeat the IVW analyses after removing each SNP. We perform the following sensitivity methods: (1) Weighted median regression, (2) Weighted mode regression, (3) MR-Egger, (4) MR pleiotropy and residual sum and outlier (MR-PRESSO) analysis, and (5) Steiger filtering. We interpret an effect as reliable when the results of the sensitivity tests are consistently in the same direction as the IVW results. More information on the sensitivity methods is found in the Supplementary Methods.

#### Multivariable MR

We use the MVMR r-package (Sanderson, Spiller, & Bowden, 2021) to perform multivariable MR (MVMR) where we predict the psychiatric outcomes using all combinations of (1) a smoking phenotype and (2) volume of a brain area. All harmonized gene–exposure and gene–outcome associations for each trait-combination selected as instruments for the exposures are included in these analyses. Since we use summary statistics with non-overlapping samples, the pairwise covariance between the SNPs is set at zero. With respect to instrument strength, the instruments are required to be strongly associated with the exposure, conditioning on the remaining exposures. This strength is quantified using a conditional *F*-statistic, with a threshold of *F* > 10. Finally, we test for heterogeneity using a modified form of Cochran’s Q statistic.

### Interpretation of results

**Figure 2.**
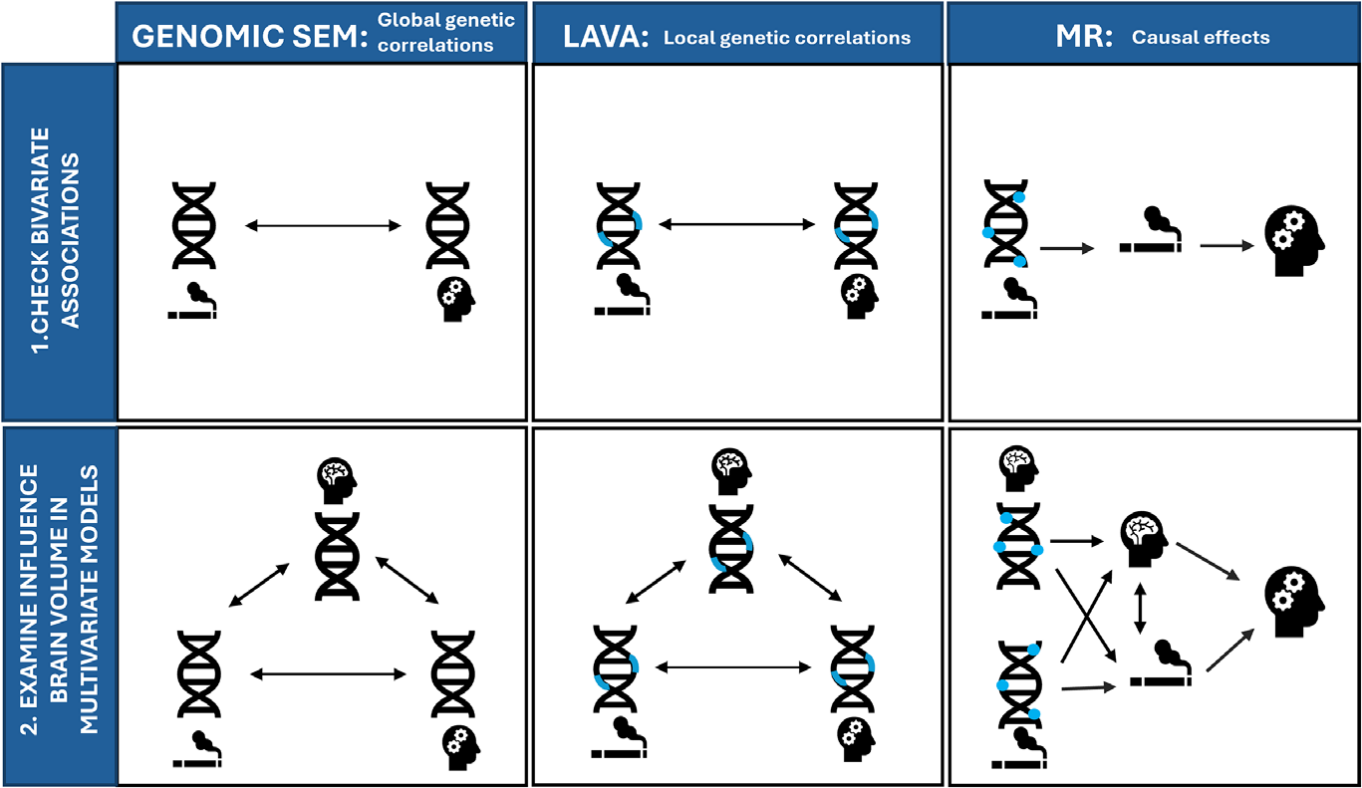
Overview of all included methods for examining (1) bivariate associations between smoking and psychiatric disorders (top row) and (2) multivariate models where we examine the influence of brain volume on these associations (bottom row). Genomic SEM = Genomic Structural Equation Model, LAVA = Local (co)Variant Association Analysis, MR = Mendelian Randomization. Blue rectangles indicate local regions, and blue circles indicate individual genetic variants associated with smoking (indicated with a cigarette pictogram), or brain volume (indicated by the brain pictogram).

We interpret *p*-values as the strength of evidence for our hypotheses, where *p* > .05 indicates extremely weak, *p* = .01–.05 indicates weak, *p* = .01–.005 indicates moderate, and *p* < .005 indicates strong evidence based on the broad interpretation of *p*-values described by Sterne and Davey Smith (Sterne & Smith, 2001). An overview of all analyses is displayed in Figure 2.

## Results

### Genomic SEM

**Figure 3.**
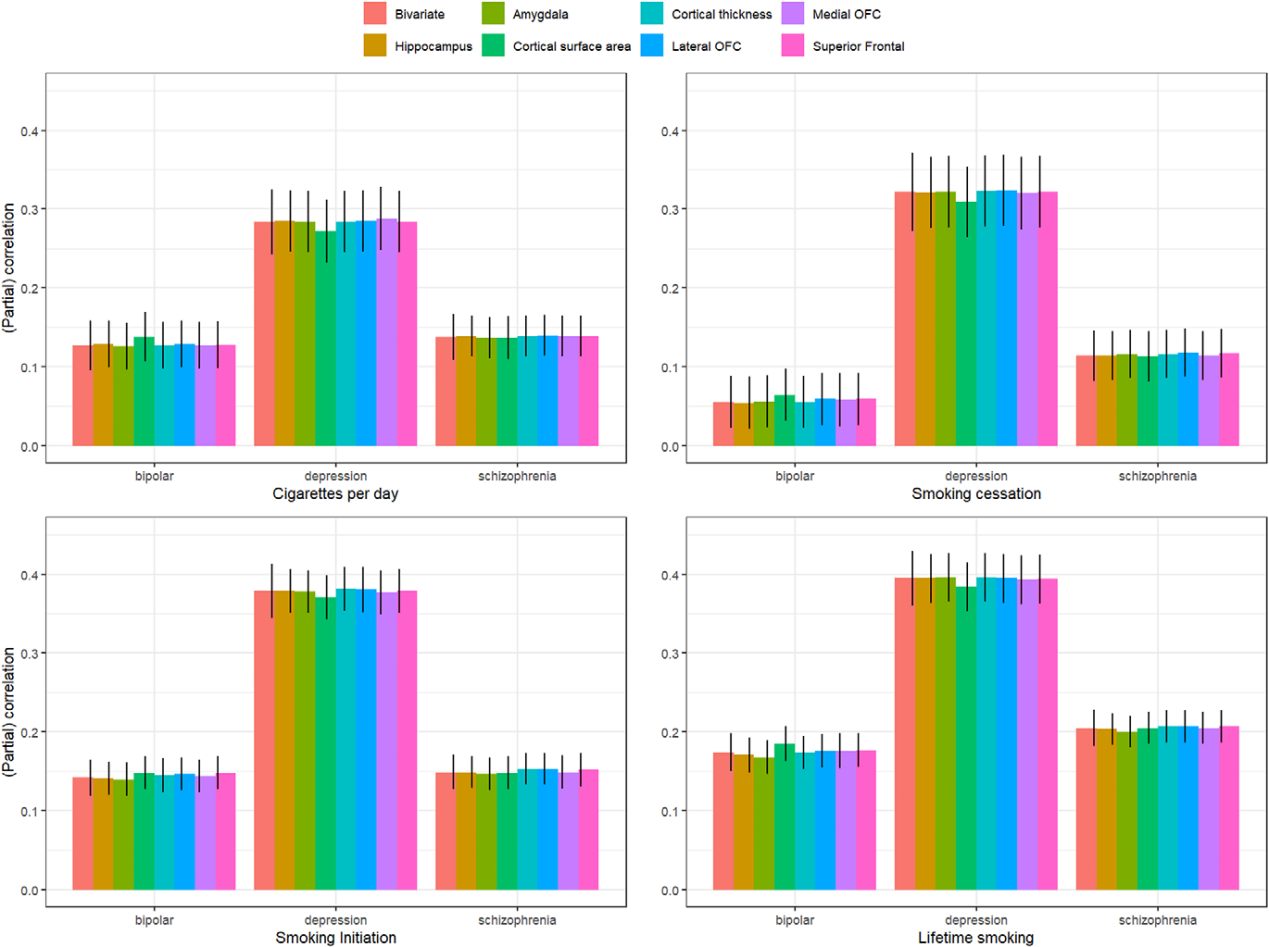
Bivariate genetic correlations between the four smoking phenotypes and three psychiatric disorders and partial correlations controlling for the different brain phenotypes.

Bivariate genetic correlations between all traits are found in Supplementary Table S1 and Supplementary Figure S1. The smoking phenotypes were positively genetically correlated with bipolar disorder (*r_g_* ranging between .055 [smoking cessation] and .174 [lifetime smoking]), major depression (*r_g_* ranging between .285 [cigarettes per day] .395 [lifetime smoking]), and schizophrenia (*r_g_* ranging between .114 [smoking cessation] and .205 [lifetime smoking]). Adjusting for the brain phenotypes did not have a statistically significant influence on associations between smoking phenotypes and psychiatric outcomes (Figure 3, Supplementary Table S2).

We found weak evidence for a mediating role (*p* < .05) of cortical surface area in the association between cigarettes per day and major depression (mediation effect = .013 (4.5%), *p* = .023), smoking cessation and major depression (mediation effect = .012 (3.7%), *p* = .035), lifetime smoking and major depression (mediation effect = .011 (2.7%), *p* = .034), and lifetime smoking and bipolar disorder (mediation effect = −.011 (−6.3%), *p* = .040).

### LAVA

**Figure 4.**
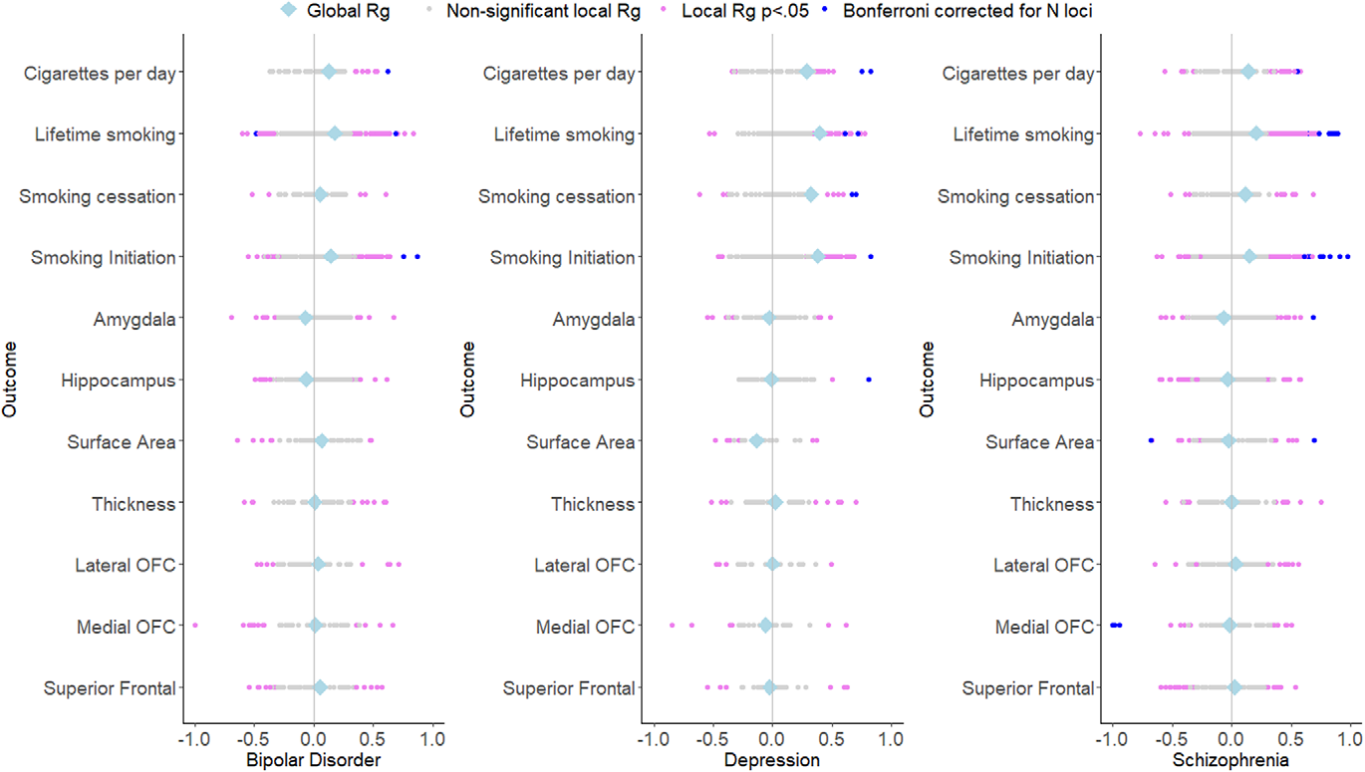
Local and global genetic correlations of the psychiatric outcomes with smoking and brain volume phenotypes.

There were 1,585 combinations where both a smoking variable and a psychiatric disorder exhibited significant univariate signal and 1,478 combinations where both a brain volume measure and a psychiatric disorder exhibited significant univariate signal (across *N* regions: 758). Across the 758 regions with a univariate signal for two or more variables, there were 43 significant local genetic correlations between smoking variables and psychiatric disorders (all but one of the associations were positive, i.e. higher levels of smoking were associated with a higher chance of psychiatric disease) and 8 significant local genetic correlations between brain measures and psychiatric disorders (3 positive, 5 negative) (see Figure 4 and Supplementary Tables S3–S4). Two regions showed significant multivariate signal: for both, there was significant bivariate signal between schizophrenia and both a smoking measure and brain volume.

One of these regions (between 3:186602046 and 3:187939199) showed strong genetic correlations of schizophrenia with lifetime smoking (local *r_g_* = .624, *p* = 1.01×10^−5^) and amygdala volume (local *r_g_* = .686, *p* = 3.31×10^−5^). In a multivariate model including both lifetime smoking and amygdala volume as predictors of schizophrenia, evidence for local genetic correlations between lifetime smoking and schizophrenia (local *r_g_* = .302, *p* = .449) and between amygdala volume and schizophrenia (local *r_g_* = .485, *p* = .265) was extremely weak. Another region (between 6:30715007 and 6:31106493) showed significant genetic correlations between schizophrenia and lifetime smoking (local *r_g_* = .831, *p* = 1.02×10^−5^) and the medial OFC (local *r_g_* = −.979, *p* = 4.27×10^−7^). In a multivariate model including lifetime smoking and medial OFC volume as predictors, evidence for local genetic correlations between lifetime smoking and schizophrenia (local *r_g_* = .186 *p* = .707) and between medial OFC volume and schizophrenia (local *r_g_* = −.857, *p* = .220) was extremely weak. However, it should be noted that for all these multivariable analyses, the confidence interval around the local genetic correlation analyses was large (i.e. ranging values below −1 to above 1 for all estimates), indicating imprecise estimates.

### Mendelian randomization

The full results from the MR analyses from smoking to psychiatric outcomes, smoking to brain volume, and brain volume to psychiatric outcomes are presented in Supplementary Tables S5–S8, S9–S12, and S13–S16, respectively.

### Smoking to psychiatric outcomes

**Figure 5.**
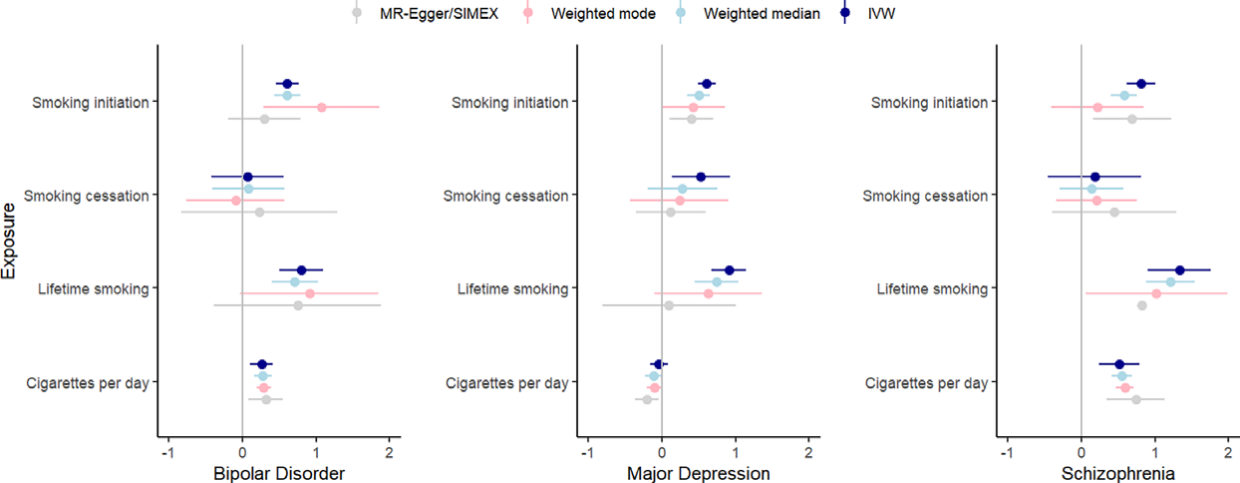
IVW, weighted median, weighted mode, and MR-Egger results for the MR analyses examining a potential causal effect of smoking phenotypes (y-axis) on psychiatric outcomes (*β* on x-axis).

Figure 5 summarizes the results for the smoking to psychiatric outcomes analyses. The F-statistic was >10 for all phenotypes (range: 24.69–70.24), indicating sufficient instrument strength. There was strong evidence for heterogeneity based on Cochran’s Q. We found strong evidence that *cigarettes per day* causally increases the risk of bipolar disorder (IVW *β* = .26, SE = .08, *p* = .002), and schizophrenia (IVW *β* = .52, SE = .14, *p* = .0002), with similar effect sizes across the weighted median, mode, and MR-Egger analyses. The MR-Egger intercept was not significantly different from zero, indicating an absence of horizontal pleiotropy. MR-PRESSO detected outliers for the cigarettes per day-schizophrenia association, but removing them did not change the results. There was moderate evidence for a causal effect of *smoking cessation* on major depression, but this effect was not replicated in the sensitivity analyses. There was strong evidence for positive causal effects of smoking initiation on bipolar disorder (IVW *β* = .61, SE = .08, *p* = 7.30×10^−14^), major depression IVW (*β* = .61, SE = .06, *p* = 6.56×10^−24^), and schizophrenia (IVW *β* = .81, SE = .10, *p* = 9.22×10^−17^), with comparable effects in weighted median and mode regression. Since the *I*^2^ statistic for these associations were below/around .60, we cannot reliably interpret the MR-Egger/SIMEX results. MR-PRESSO detected outliers, but removing them did not change the results. Finally, there was strong evidence that the liability for *lifetime smoking* causally increases risk for bipolar disorder (IVW *β* = .80, SE = .15, *p* = 1.52×10–7), major depression (IVW *β* = .91, SE = .12, *p* = 2.54×10^–13^), and schizophrenia (IVW *β* = .1.33, SE = .22, *p* = 2.29×10^–9^), with similar effect sizes in weighted median and mode regression. The MR-Egger intercept was not significantly different from zero, suggesting an absence of horizontal pleiotropy. However, the MR-Egger slope indicated smaller and non-significant effects: bipolar disorder (*β* = .75, SE = .58, *p* = .20) and major depression (*β* = .10, SE = .46, *p* = .82) and schizophrenia (*β* = .82, SE = .82, *p* = .32). MR-PRESSO detected outliers for these associations, but removing them did not impact the results. For all instruments, MR-Steiger identified SNPs more predictive of the outcome than the exposure. The identified associations were still significant when re-running the analyses without these SNPs, but the effect sizes were somewhat reduced.

### Smoking to brain volume

**Figure 6.**
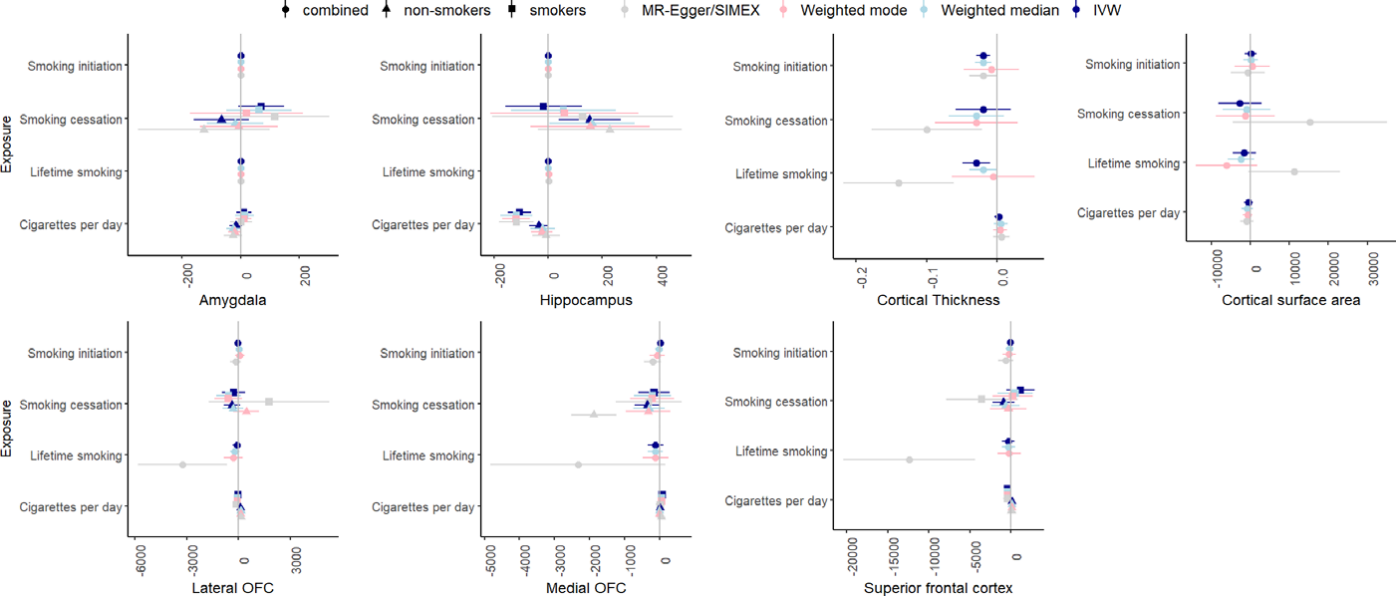
IVW, weighted median, weighted mode, and MR-Egger results for the MR analyses examining a potential causal effect of smoking phenotypes (y-axis) on brain volume (x-axis, scales differ per phenotype depending GWAS).

Figure 6 summarizes the results for the smoking to brain volume analyses. The F-statistic was >10 for all phenotypes (range: 18.83–91.12). Cochran’s Q indicated strong evidence for heterogeneity for associations between brain volumes and smoking initiation and lifetime smoking. There was strong evidence for a negative effect of cigarettes per day on hippocampal volume in smokers (IVW *β* = −106.63, SE = .22.18, *p* = 1.53×10^−6^), replicating across weighted mode (*β* = −118.29, SE = 29.96, *p* = 7.87×10^−5^), weighted median (*β* = −119.92, SE = 25.82, *p* = 3.09×10^−5^), and MR-Egger analyses (*β* = −118.87, SE = 32.59, *p* = .0007). MR-PRESSO did not identify outliers, and the Egger intercept did not deviate from zero. In addition, there was a significant negative causal effect of *smoking initiation* on cortical thickness in the IVW regression (IVW *β* = −.02, SE = .005, *p* = .0001), but this effect disappeared when removing SNPs with reverse causal effects using MR-Steiger (IVW *β* = −.005, SE = .005, *p* = .30). A significant negative effect of lifetime smoking on cortical thickness was identified (IVW *β* = −.03, SE = .01, *p* = .005), which was reduced in weighted median (*β* = −.02, SE = .01, *p* = .05) and mode (*β* = −.006, SE = .03, *p* = .83) regression, but much larger in SIMEX-corrected MR-Egger (*β* = −.14, SE = .04, *p* = .001). MR-PRESSO did not detect outliers, but the effect disappeared after Steiger filtering (*β* = −.01, SE = .008, *p* = .18).

### Brain volume to psychiatric outcomes

**Figure 7.**
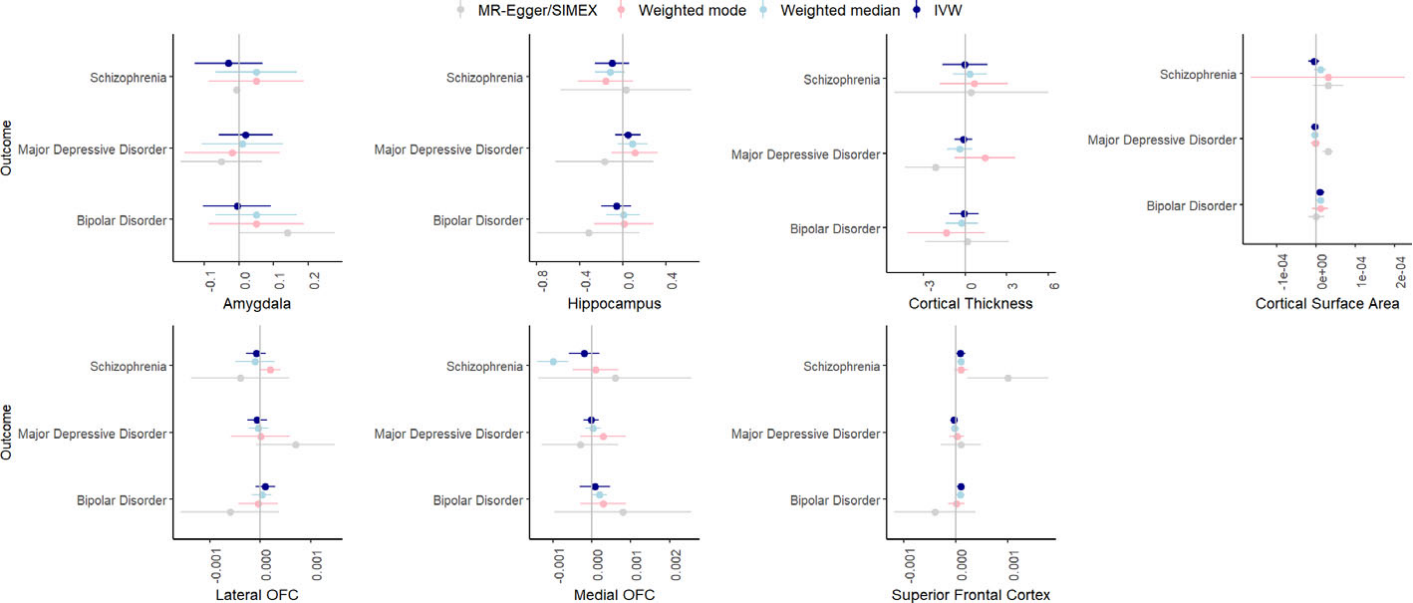
IVW, weighted median, weighted mode, and MR-Egger results for the MR analyses examining a potential causal effect of brain volume (x-axis, scale differs depending on GWAS) on psychiatric disorders (y-axis).

Figure 7 summarizes the results for the MR analyses of brain volume to psychiatric outcomes. F-statistic was higher than 10 for all phenotypes. There was some evidence for heterogeneity for most associations based on Cochran’s Q.

In the IVW analyses, there was strong evidence only for a significant, positive effect of superior frontal cortex volume on bipolar disorder (*β* = .0001, SE = .00004, *p* = .005). This effect was somewhat consistent across the weighted median (*β* = .00009, SE = .00004, *p* = .03) and weighted mode analyses (*β* = .00001, SE = .00008, *p* = .89) but could not reliably be tested with MR-Egger/Egger-SIMEX since the I^2^ was zero. Leave-one-out analyses indicated that removal of some of the SNPs led to weaker effects, and MR-PRESSO detected one outlier, removal of which resulted in a weaker association (*β* = .00009, SE = .00004, *p* = .02).

### Multivariable MR

Results for the MVMR analyses are found in Supplementary Tables S17–S20. We found lower conditional F-statistics for both exposure types (smoking and brain volumes) than in the univariable analyses. In the analyses including cigarettes per day, smoking initiation, or lifetime smoking, the conditional F-statistic for volume of different brain areas was around/below 10, indicating weak instrument bias. In the models including smoking cessation, the conditional F was around/below 10 for smoking cessation. We also found high Q-values, indicating heterogeneity, but these might be inflated due to weak instrument bias. Across the models, smoking exposures had similarly sized effects on the psychiatric outcomes as in the univariable MR analyses. However, we found somewhat smaller effects of smoking initiation on bipolar disorder and schizophrenia in models, including volume of the lateral OFC, medial OFC, and superior frontal cortex (bipolar: multivariable *β* between .32 and .43, schizophrenia: multivariable *β* between .45 and .65). In addition, we found somewhat larger effects of smoking cessation on major depression in models, including volume of the lateral OFC, medial OFC, and superior frontal cortex (multivariate *β* between 1.11 and 1.52).

## Discussion

We investigated whether causal effects of smoking on the risk of depressive disorder, bipolar disorder, schizophrenia are (partially) mediated by brain volume of different brain areas (amygdala, hippocampus, lateral and medial OFC, superior frontal cortex), and global brain volume measures (cortical thickness and surface area). By using genetic methods that employ summary statistics, we circumvented the issue of the absence of a dataset with sufficient sample size on all measures. In general, we find small-to-medium genetic correlations between smoking measures and psychiatric disorders, and no to small genetic correlations between smoking and brain measures. In addition, we find evidence for causal effects of the different smoking measures on all psychiatric outcomes, evidence for a few causal effects of smoking on brain volume (hippocampal volume and cortical thickness) but no causal effects of brain volume measures on psychiatric disease. All in all, there was no evidence for a mediating role of the included brain volumes in smoking to psychiatric disorder associations.

First, we examined genetic correlations using genomic SEM. Similar to previous work (Jang et al., 2022), we found small-to-moderate positive genetic correlations between smoking and psychiatric disorders, with the smallest associations with bipolar disorder and the largest associations with depressive disorder. Adjusting for brain volume did not affect these associations. We generally found a lack of genetic correlations between brain volume and psychiatric disorders, in line with Liu and colleagues (Liu, Smit, Abdellaoui, van Wingen, & Verweij, 2023), who found that psychiatric disorders are more strongly correlated to brain function than structure. We were also interested in zooming into local associations using LAVA, as local genetic correlations in opposite directions cancel each other out in global correlations. We found 43 significant local genetic correlations between smoking and psychiatric outcomes, of which only one was negative, confirming the positive association (i.e. higher levels of smoking associated with higher chances of psychiatric outcomes) on a local level. Local associations between brain volume and psychiatric disease were less uniform, with three positive and five negative associations. There were two regions with significant bivariate signal between schizophrenia and both a smoking measure and brain volume. One of the regions is located in the MHC region, which is extremely pleiotropic for immune- and health-related traits (Watanabe et al., 2019). The other is located on chromosome 3 in a region spanning multiple genes (Supplementary Table S20). Finally, to examine causality, we performed MR. We replicate causal effects of smoking on the psychiatric disorders (Treur et al., 2021) and of smoking on amygdala and hippocampal volume (Logtenberg et al., 2022). We also found effects of smoking initiation and lifetime smoking on cortical thickness, but these disappear after Steiger filtering. We did not find significant effects of brain volume on psychiatric outcomes. In addition, the multivariate models generally did not reduce associations between smoking and psychiatric disease, with a few exceptions (but these analyses had low power). Of note, we interpret *p*-values as the strength of the evidence for the hypotheses, where *p* > .05 indicates extremely weak, and *p* < .005 indicates strong evidence ((Sterne & Smith, 2001), instead of conducting a Bonferroni (or similar) correction where we correct for the number of tests. Since the tests we perform are not necessarily independent (e.g. multiple measures of correlated smoking exposures) and the associations tested are based on findings from previous research in the same or similar datasets (e.g. our previous MR work identifying the smoking – subcortical brain volume associations in the same data), we believed such a correction was too strict. While others may have performed more strict corrections, this would not have changed our conclusion of an absence of mediation of the included brain volume measures.

These findings should be interpreted in light of some limitations. First, whereas the GWASs included in this paper are large and had sufficient instrument strength, they still index only a small percentage of the variation in their respective phenotypes and are the result of a meta-analysis of multiple cohorts, potentially leading to more noise. In addition, the summary statistics for the brain volume measures were based on much smaller samples than the other phenotypes and thus held lower power. However, this would have the largest effect on the standard error rather than the (low) point estimates found for genetic correlations with brain volume. In addition, the F-statistic of the MR analyses indicated sufficient instrument strength, albeit lower than for the smoking instruments. In addition, we did not have smoking-stratified summary statistics for the psychiatric outcomes. This means that samples on which these GWASs are based are a mix of smokers and non-smokers, and that the effects in smokers must thus be strong enough to counterbalance that the association cannot be observed in the non-smokers. While we did identify effects of smoking on psychiatric outcomes using unstratified summary statistics, these effects are likely an underestimate. With respect to the sample, all summary statistics were based on European ancestry samples, meaning that the results do not necessarily generalize to other ancestries. In addition, there might be selection bias, as individuals who participate in genetic studies are not always representative of the general population. This volunteer bias has previously been demonstrated for the UK Biobank, where individuals were found to be more likely to be older, healthier and of higher socioeconomic status (van Alten, Domingue, Faul, Galama, & Marees, 2024). Our GWASs in UKB thus suffer from this limitation as well. Finally, MR is a powerful tool for examining causality when an RCT is not practical or feasible, but is dependent on strict assumptions which may have been (partly) violated. In addition, there are important limitations to the interpretation of the effects of an MR analysis, due to the effects reflecting lifetime genetic liability. We therefore only interpret the effects of our MR analyses in terms of the absence or presence of a causal effect, and not in terms of the magnitude of that effect. In order to achieve more reliable evidence on the mechanisms that explain causality between smoking and psychiatric disease, the MR evidence should be triangulated with evidence from different study types, such as longitudinal studies.

Given these limitations, we cannot exclude the possibility that brain volume explains a part of the association between smoking and psychiatric disorders, but, if present, this would likely be a very small part. Importantly, our results only pertain to the included brain areas, which were selected based on prior research. Similarly, we chose to focus on psychiatric outcomes previously linked to smoking in MR research (Treur et al., 2021), but this does not exclude the possibility of other psychiatric outcomes being linked to smoking. In addition, there may be an effect, but this effect might depend on the age of smoking initiation, in that we would only observe an effect if smoking was initiated when the brain is still developing. At the same time, there may also be a role for brain volume, but in a different direction then examined here. A likely hypothesis is that individuals with psychiatric disorders are more prone to smoke, and that this eventually results in lower brain volume. However, since we were interested in explaining the effect of smoking on psychiatric disease, and not vice versa, this hypothesis was not explored. In addition, it is likely that many mechanisms are simultaneously at play. As mentioned in the introduction, there are many ways in which the brain may play a role in the effect of smoking on psychiatric disease, such as through alterations in functional connectivity. For example, research suggests that smokers have lower connectivity than non-smokers in the default mode network (Weiland, Sabbineni, Calhoun, Welsh, & Hutchison, 2015), a network that also seems to have abnormal connectivity in individuals with psychiatric disorders (Doucet et al., 2020). These functional alterations take place on a more rapid timescale than structural changes, making this an interesting potential pathway for future research. Finally, there may also be non-biological mechanisms and confounders (e.g. physical exercise, alcohol use) influencing these associations that are not captured in the genetic correlations. While not explicitly modeled in the genetic correlation analyses, if these are downstream consequences of smoking, genetic correlations would partly capture these effects through vertical pleiotropic effects. If these factors are (heritable) confounders of the smoking-psychiatric disease association, they would likely be captured by the genetic correlations and result in inflated correlations. However, for the MR analyses, unless these confounders are associated with our genetic instruments, these confounders should not inflate our MR findings. A confounder that is important to mention is SES, as this is a potential example of this situation. Previous research has found that SES-associated genetic variation influences GWAS results, potentially due to geographical clustering of SES (Marees et al., 2021). This potentially violates the second MR assumption of independence and should be examined in future research, potentially using MVMR.

To conclude, we present the first study testing whether structural brain measures mediate the link between smoking and psychiatric outcomes. We use genetic methods that do not require individual-level data to study global and local genetic associations and assess whether identified associations reflect causality. We do not find evidence for a mediating role of the included brain areas but note that our findings are limited by sample characteristics of the GWASs. Further research is needed to explore other potential mechanisms, and genetic methods such as the ones used here could aid these explorations.

## Supporting information

van de Weijer et al. supplementary material van de Weijer et al. supplementary material

## Data Availability

The summary statistics used for these analyses are publicly available. Summary statistics including 23andme data can be requested at 23andme. We would like to thank the research participants and employees of 23andMe, Inc. and UK Biobank for making this work possible. The GWASs we ran on brain volume measures were ran using UKB data (Application Number 40310) and summary statistics can be requested by contacting the corresponding author.

## Supplementary material

The supplementary material for this article can be found at http://doi.org/10.1017/S0033291725100561.
