# Supplementary material for: The causal effect of smoking on psychiatric disorders: an examination of brain volume as a potential pathway
Source: Psychol Med. 2025 Jun 24;55:e171. doi: 10.1017/S0033291725100561 (PMC12201961; doi:10.1017/S0033291725100561)
Supplement: van de Weijer et al. supplementary material [file S0033291725100561sup001.zip › Supplementary Figures.docx]

**Supplementary Figures**


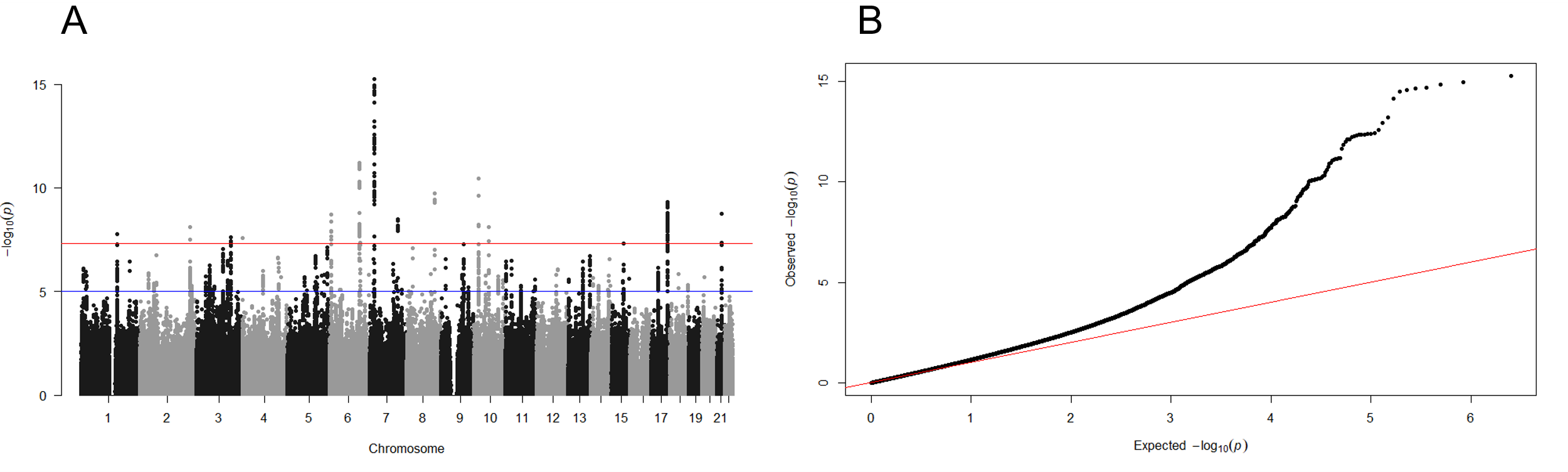


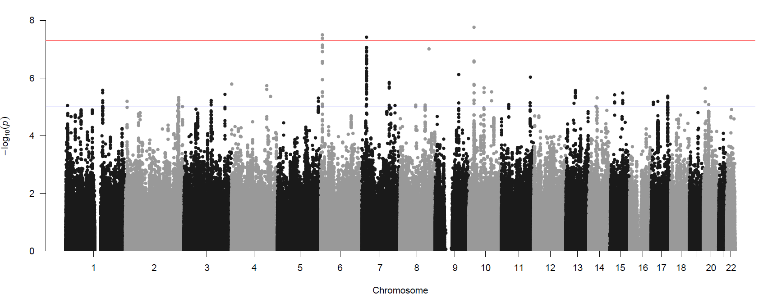

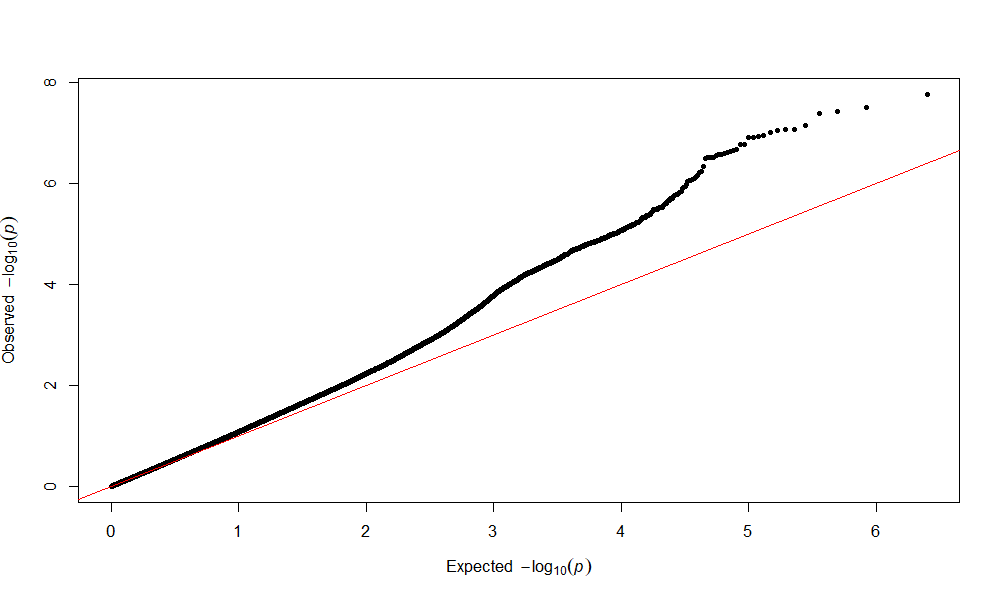
*Supplementary Figure S1.* Manhattan plot (left) and Q-Q plot (right) for the GWAS of lateral orbitofrontal cortex volume.


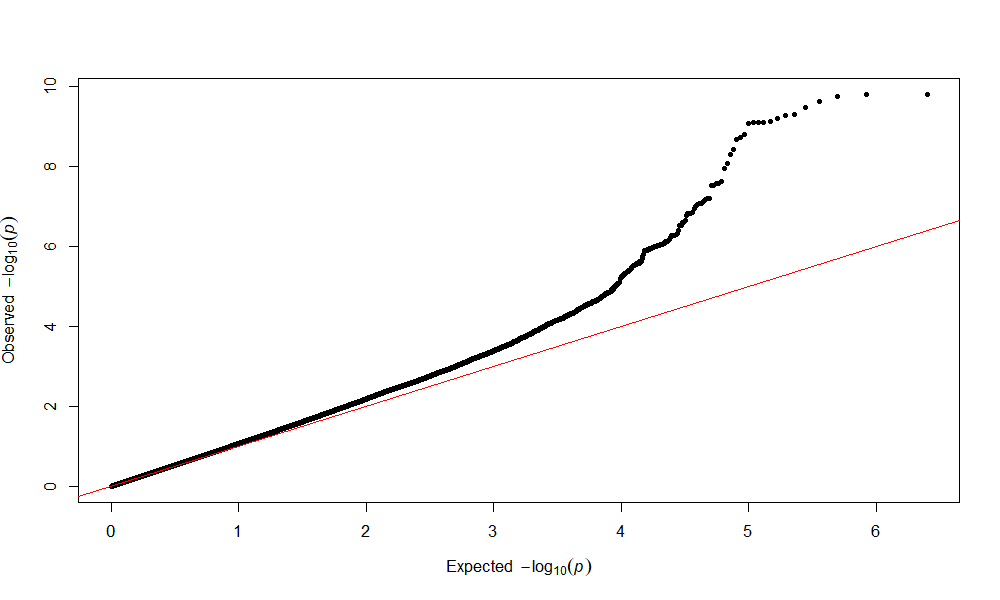
*Supplementary Figure S2.* Manhattan plot (left) and Q-Q plot (right) for the GWAS of lateral orbitofrontal cortex volume in smokers


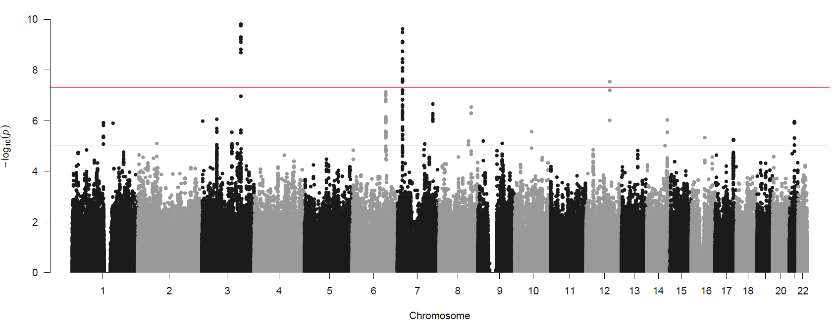


*Supplementary Figure S3.* Manhattan plot (left) and Q-Q plot (right) for the GWAS of lateral orbitofrontal cortex volume in non-smokers.


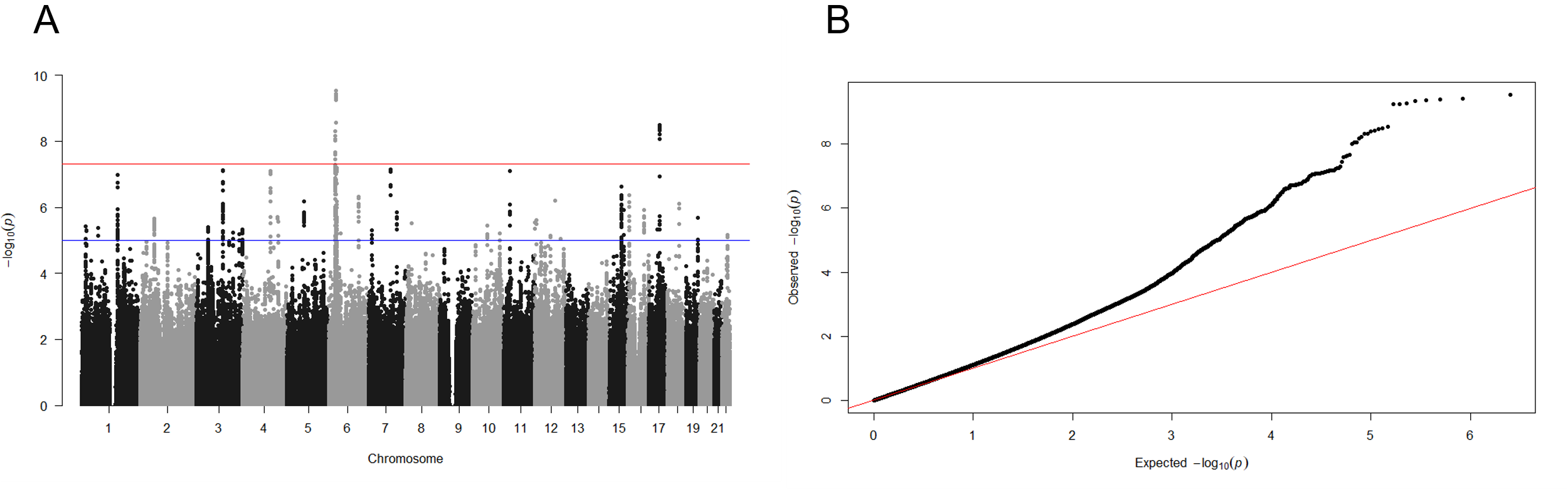


*
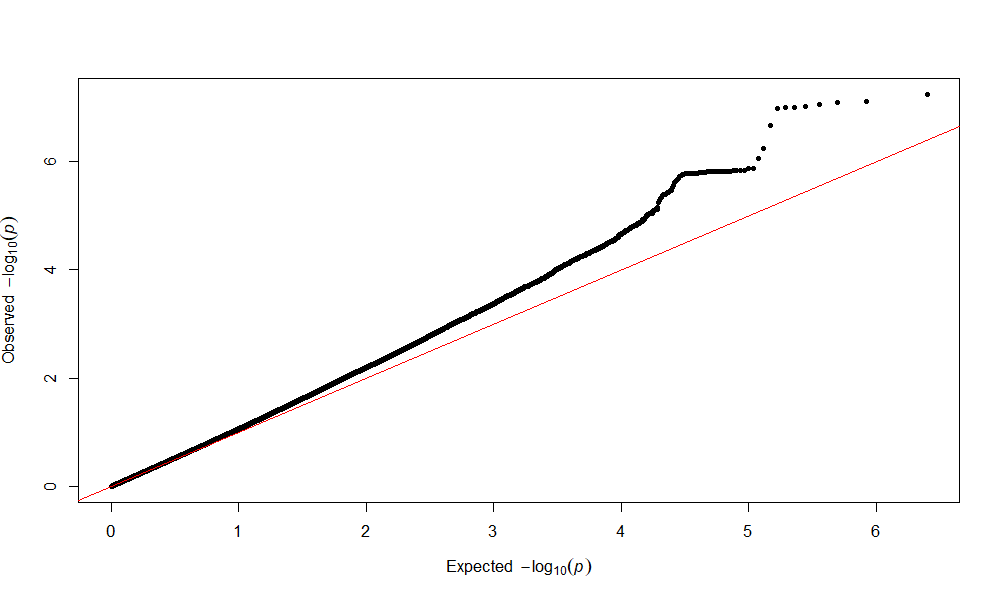
Supplementary Figure S4* Manhattan plot (left) and Q-Q plot (right) for the GWAS of medial orbitofrontal cortex volume.


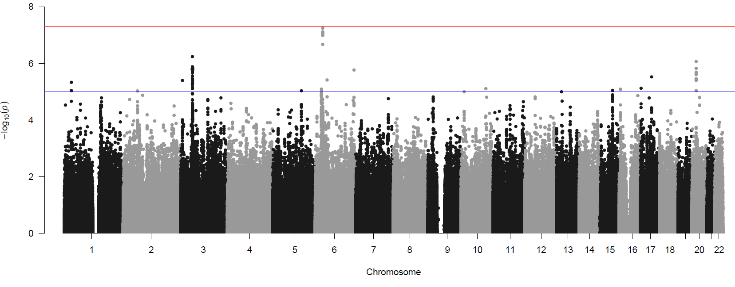


*Supplementary Figure S5.* Manhattan plot (left) and Q-Q plot (right) for the GWAS of medial orbitofrontal cortex volume in smokers.


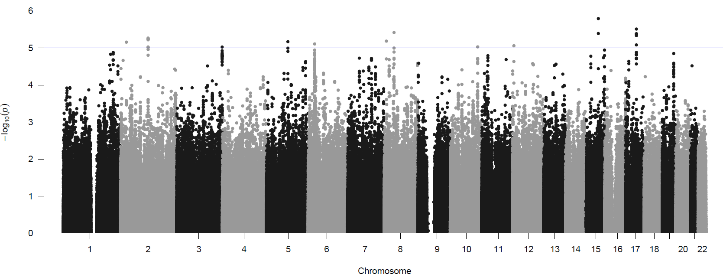

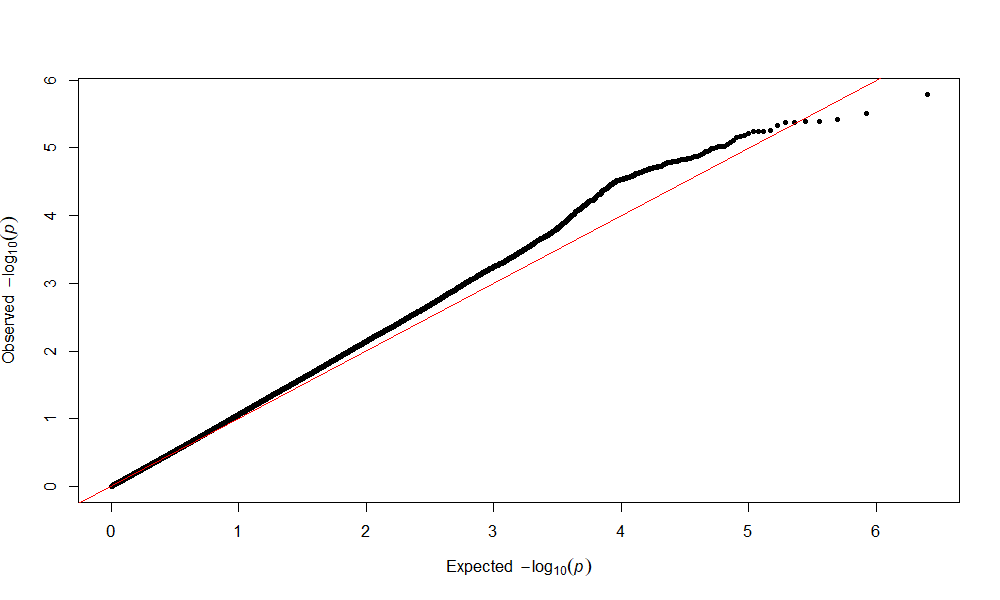


*Supplementary Figure S6.* Manhattan plot (left) and Q-Q plot (right) for the GWAS of medial orbitofrontal cortex volume in non-smokers.


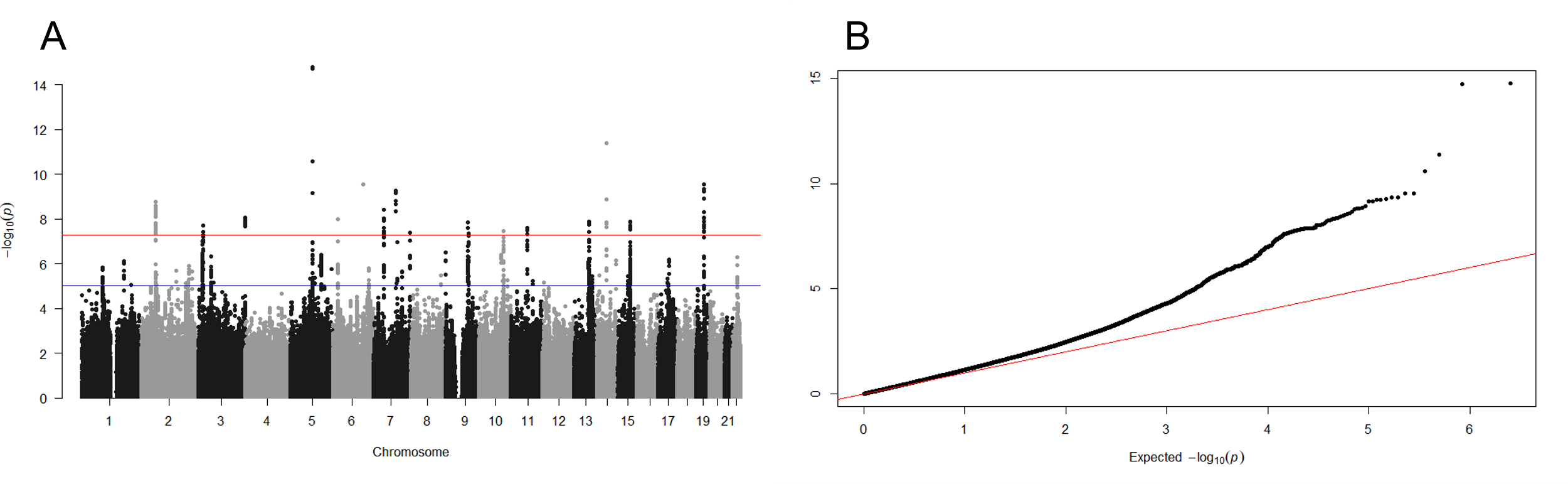


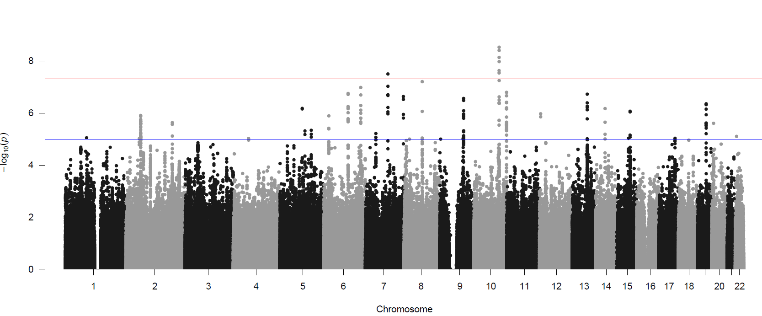
*Supplementary Figure S7.* Manhattan plot (left) and Q-Q plot (right) for the GWAS of superior frontal cortex volume.


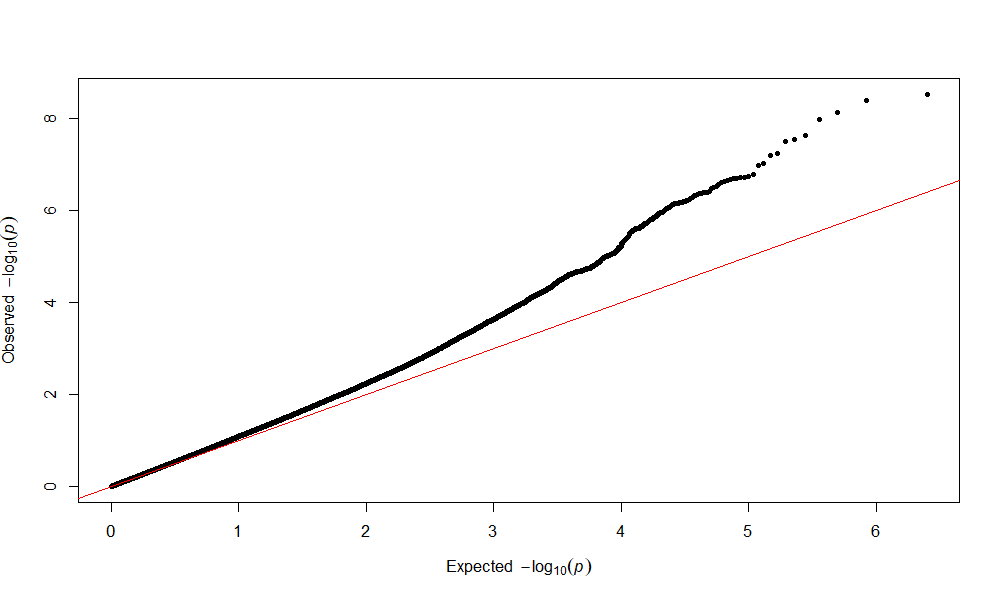


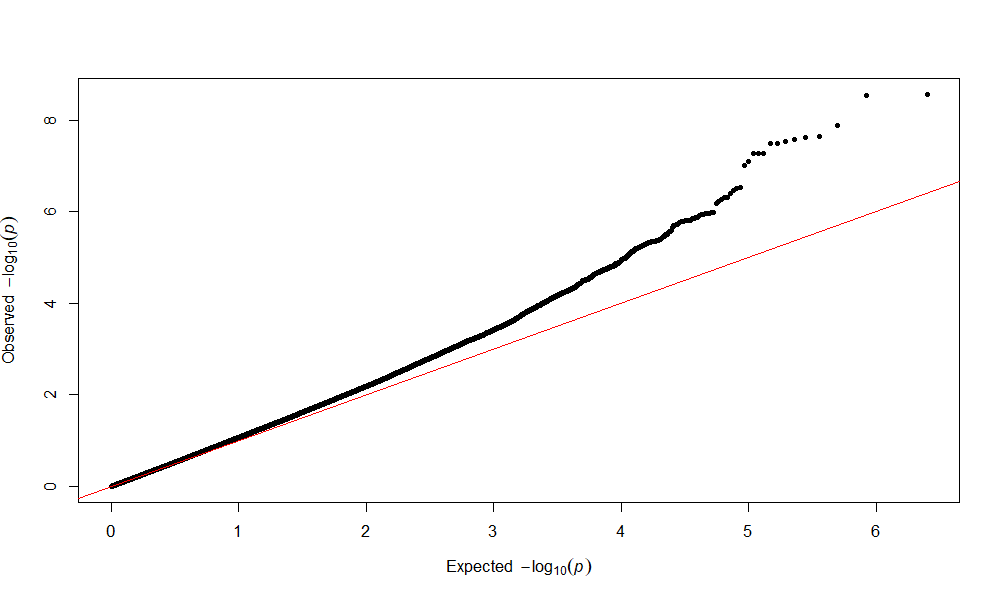
*Supplementary Figure S8.* Manhattan plot (left) and Q-Q plot (right) for the GWAS of superior frontal cortex volume in smokers.


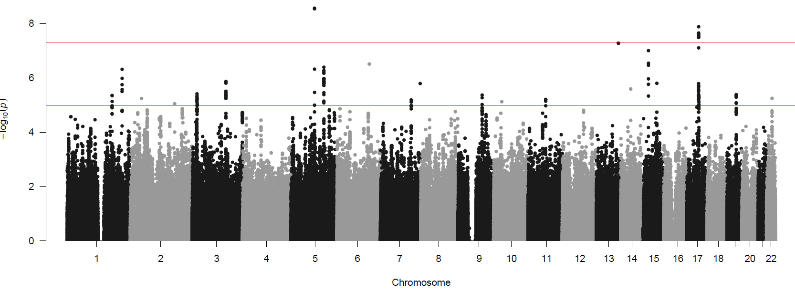


*Supplementary Figure S9.* Manhattan plot (left) and Q-Q plot (right) for the GWAS of superior frontal cortex volume in non-smokers.
