# Supplementary material for: The causal effect of smoking on psychiatric disorders: an examination of brain volume as a potential pathway
Source: Psychol Med. 2025 Jun 24;55:e171. doi: 10.1017/S0033291725100561 (PMC12201961; doi:10.1017/S0033291725100561)
Supplement: van de Weijer et al. supplementary material [file S0033291725100561sup001.zip › Supplementary Methods.docx]

**Supplementary Methods**

*Deviations from the pre-registration*

1. We pre-registered using depression summary statistics from Levey and colleagues, but ended up using summary statistics by Wray and colleagues since Levey et al was not publicly available.
2. We pre-registered using GSMR as a sensitivity analysis but eventually did not run this analysis as we already included several methods that account for and examine the same type of bias (pleiotropy and invalid instruments
3. We pre-registered repeating analyses with within-family GWAS where possible, but given the absence of well-powered within-family GWASs for the relevant traits, did not perform these analyses
4. We pre-registered correcting for SES in our analyses to rule out that the mediating effect was (partly) due to SES. However, since we did not find any evidence for a mediating effect, these analyses were redundant.

*Genome-wide association studies UKB*

We performed genome-wide association studies (GWAS) for volume of three brain areas as these were not publicly available (they were only available for left and right volume separately). We first calculated average brain volume of the left and right hemisphere volume of: the lateral orbitofrontal cortex (UKB ID 26799 and 26900), the medial orbitofrontal cortex (UKB ID 26801 and 26902) and the superior frontal cortex (UKB ID 26815 and 26916). We performed the GWASs in a subset of UKB participants of European ancestry, as determined by principal component analysis (PCA) in GCTA. SNP quality control procedures included filtering out SNPs with a MAF <.01, missingness >.05, and HWE p<10^-10^. A detailed description of the quality control procedures and data processing can be found in Abdellaoui et al., 2019 (1). In total, 36,792 individuals were included in the analyses. The GWASs were run using the linear mixed model function in fastGWA(2), using the first 25 genomic principal components, genotype array, sex, age, age^2^, and total brain volume (UKB ID 26521) as covariates. We additionally re-ran these GWASs for smokers (n=13,645) and non-smokers (n=22,798) separately, based on smoking status (UKB ID 20116). Supplementary figures 1-8 display Manhattan and Q-Q plots for all these GWASs.

*Mendelian Randomization Sensitivity Methods*

Weighted median regression (3), implemented in the TwoSampleMR r-package (4), produces a reliable causal estimate if <50% of the total weight of the genetic instrument comes from biased SNPs (that do not satisfy MR assumptions). Weighted mode regression, implemented in the TwoSampleMR r-package (4), clusters the selected SNPs and selects the cluster with the largest weight as the causal estimate, which is unbiased if the largest number of similar individual causal effects come from valid instruments (5). MR-Egger tests for horizontal pleiotropy (i.e., when SNPs are directly associated with both exposure and outcome) by freely estimating the intercept (6). When the intercept deviates from zero, this indicates the presence of horizontal pleiotropy. MR-Egger relies on two assumptions: 1) the Instrument Strength Independent of Direct Effect (InSIDE) which assumes that the strength of the SNP-exposure association is uncorrelated with the SNP-outcome association, and 2) the no measurement error (NOME) assumption, which assumes that the instruments are sufficiently strong. We test the NOME assumption by calculating the I^2^ (7). If this statistic is >.9, we assume that bias due to NOME violations is unlikely. When the I^2^ is <.9, we correct for bias using simulation extrapolation (SIMEX) MR-Egger (8). When the I^2^ is <.6, Egger/SIMEX results are unlikely to be reliable. MR-PRESSO (9) consists of three tests: 1) the MR-PRESSO global test that detects horizontal pleiotropy by comparing the observed residual sum of squares (SS) with the expected residual SS for each SNP, 2) the MR-PRESSO outlier correcting for horizontal pleiotropy via outlier removal, and 3) the MR-PRESSO distortion test which tests distortion in the causal estimates before and after outlier removal. The SNPs are selected through a procedure called HEIDI-filtering, which removes variants with pleiotropic effects. Lastly, Steiger filtering (10) is used to correct for reverse causality by testing if SNPs explain a larger amount of variance in the outcome than the exposure.

1. Abdellaoui A, Hugh-Jones D, Yengo L, Kemper KE, Nivard MG, Veul L, et al. Genetic correlates of social stratification in Great Britain. Nature Human Behaviour. 2019;3(12):1332-42. doi: 10.1038/s41562-019-0757-5.

2. Jiang L, Zheng Z, Qi T, Kemper KE, Wray NR, Visscher PM, Yang J. A resource-efficient tool for mixed model association analysis of large-scale data. Nature Genetics. 2019;51(12):1749-55. doi: 10.1038/s41588-019-0530-8.

3. Bowden J, Davey Smith G, Haycock PC, Burgess S. Consistent Estimation in Mendelian Randomization with Some Invalid Instruments Using a Weighted Median Estimator. Genetic Epidemiology. 2016;40(4):304-14. doi: 10.1002/gepi.21965.

4. Hemani G, Zheng J, Elsworth B, Wade KH, Haberland V, Baird D, et al. The MR-Base platform supports systematic causal inference across the human phenome. eLife. 2018;7:e34408. doi: 10.7554/eLife.34408.

5. Hartwig FPDS, George; Bowden, Jack. Robust inference in two-sample Mendelian randomisation via the zero modal pleiotropy assumption. International Journal of Epidemiology. 2017;46(6):1985-98.

6. Bowden JDS, George; Burgess, Stephen. Mendelian randomization with invalid instruments: effect estimation and bias detection through Egger regression. International Journal of Epidemiology. 2015;44(2):512-25.

7. Bowden J, Del Greco M F, Minelli C, Davey Smith G, Sheehan NA, Thompson JR. Assessing the suitability of summary data for two-sample Mendelian randomization analyses using MR-Egger regression: the role of the I 2 statistic. International journal of epidemiology. 2016;45(6):1961-74.

8. Barry C, Liu J, Richmond R, Rutter MK, Lawlor DA, Dudbridge F, Bowden J. Exploiting collider bias to apply two-sample summary data Mendelian randomization methods to one-sample individual level data. PLoS Genetics. 2021;17(8):e1009703.

9. Verbanck M, Chen C-Y, Neale B, Do R. Detection of widespread horizontal pleiotropy in causal relationships inferred from Mendelian randomization between complex traits and diseases. Nature Genetics. 2018;50(5):693-8. doi: 10.1038/s41588-018-0099-7.

10. Hemani G, Tilling K, Davey Smith G. Orienting the causal relationship between imprecisely measured traits using GWAS summary data. PLOS Genetics. 2017;13(11):e1007081. doi: 10.1371/journal.pgen.1007081.
